# The Pasteur’s Dictum: Nitrogen Promotes Growth and Oxygen Reduces the Need for Sugar

**DOI:** 10.3389/fonc.2014.00051

**Published:** 2014-03-17

**Authors:** Lakshmipathi Vadlakonda, V. D. K. Reddy, Mukesh Pasupuleti, Pallu Reddanna

**Affiliations:** ^1^Cell Biology and Enzymology, Department of Zoology, Kakatiya University, Warangal, India; ^2^Department of Animal Sciences, School of Life Sciences, University of Hyderabad, Hyderabad, India; ^3^SRM Research Institute, Sri Ramaswamy Memorial University, Chennai, India; ^4^National Institute of Animal Biotechnology, Hyderabad, India

**Keywords:** Pasteur, nitrogen, glucose, mTOR, IIS, growth

In our previous article (1), we presented a unifying principle in the hypotheses of Pasteur, Warburg, and Crabtree that “*an inverse relation exists between glucose uptake and respiration*.” Warburg’s hypothesis laid emphasis on glucose metabolism and damaged respiration (mitochondria) for growth of cells in cancer (2). Pasteur recognized that yeast can grow only if ammonium tartrate (nitrogen source) is available, whether oxygen is available or not (3) and ammonium is transformed into a “complex albuminoid” (protein) material. Growth is faster in the presence of oxygen and for producing one unit of mass, yeast requires only 1/15th of glucose when compared to that in the absence of oxygen (4–10 parts as against 60–80 parts). Since the ratio of ATP produced per one glucose molecule in glycolysis/oxidative phosphorylation (OXPHOS) is 1:15, the energy consumed per unit of growth of yeast remains same (60X1 and 4X15 ATP). We call this: “*the Pasteur’s dictum*,” which is different from the “*Pasteur effect*” introduced by Warburg. Lagunas et al. (4) in early 80s reported that Pasteur effect is observed only in resting cells in the absence of nitrogen, but not in growing cells. Warburg’s hypothesis relies on energetics of glucose metabolism and damaged respiration but ignores Pasteur’s demonstration of nitrogen as the primary requirement for triggering growth. It also ignores the anabolic functions of mitochondria (5, 6) and the role of glutamine, another key nutrient avidly consumed by actively proliferating cells (7, 8). We present in this article, a hypothesis that activation of complexes of mechanistic target of rapamycin (mTORC1 and C2) by amino acids (nitrogen source) is the molecular explanation of the “Pasteur’s dictum.” Amino acids activate both mTORC1 and C2 independent of insulin or growth factor signaling (IIS). Amino acids are also required for activation of mTORC1 in IIS dependent pathway.

## Mechanistic Target of Rapamycin as the HUB of Nutrient Sensing

Pasteur’s work demonstrates that the quality of nutrients is important for cell growth. PI3K–Akt–mTOR signaling is recognized as a key player in uptake of nutrients (9). mTOR is a multi-protein complex and exists in two complexes, mTORC1 and mTORC2. While mLST8 and raptor are key components of mTORC1, mLST8, rictor, mSIN1 are required for mTORC2 assembly and activity [reviewed in Ref. (10, 11)]. Nutrients, growth factors, and oxygen were shown to activate mTORC1 (12); the upstream regulators of mTORC2 are less understood. We have earlier shown that a reciprocal relation exists between mTORC1 and mTORC2, which is intertwined with Akt phosphorylations (13). While mTORC2 is an upstream kinase of Akt S473 phosphorylation (14), mTORC1 is the downstream effector of Akt T308 phosphorylated state. Activated mTORC1 inhibits mTORC2 assembly (see below).

## Activation of mTOR Complexes by Amino Acids is the Modern Explanation of “Pasteur’s Dictum”

Amino acids were shown to activate mTORC1, independent of the growth factor signaling (15–17) and are also required for mTORC1 activity even under growth factor mediated conditions (18). Recent reports suggest that mTORC2 can also be activated by amino acids (19, 20). Tato et al. demonstrated that starvation and culture conditions influence activation of either mTORC1 or mTORC2 by amino acids (20). Rosario et al. demonstrated that in primary human placental cells, activation of amino acid transporters requires both mTORC1 and mTORC2 (19); localization of amino acid transporters is Tor2-independent in fission yeast (21). This indicates that mTORC1 could be the primary requirement for amino acid uptake. Activation of mTORC1 appears to precede mTORC2, which is in tune with its role in protein translation. But, Akt stability during translation requires mTORC2 (22); it later phosphorylates Akt at S473 (14).

**Figure 1:**
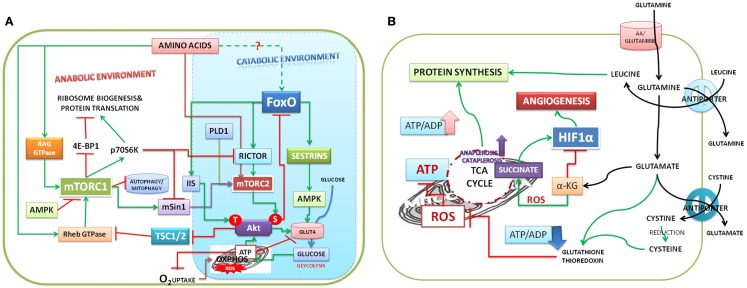
**(A)** Model depicting amino acids that trigger the downstream signals: amino acids activate mTORC1 and mTORC2 and protein biosynthesis. Both mTORC1 and mTORC2 cooperate with each other in stabilizing Akt translation. mTORC2 phosphorylates AktS473 and promotes glucose uptake. Akt S473 phosphorylates and causes nuclear exclusion of FoxO and promotes oxidative phosphorylation and ATP production (see Ref. 73). IIS activates PDPK1 (3-phosphoinositide dependent protein kinase-1), which phosphorylates the Akt at T308. Akt phosphorylations are stabilized by high ATP/ADP ratio. Akt under IIS activates mTORC1 to promote the anabolic environment. High ATP/ADP ratio inhibits uptake of glucose by phosphorylation of glucose transporter. S6K, one of the targets of mTORC1, inhibits mTORC2 by phosphorylating both rictor and mSin1. Inhibition of autophagy and mitophagy by mTORC1 shifts the mitochondrial function from ATP production to supply of amino acids, citrate for biosynthesis of proteins/lipids. **(B)** Model depicting the role of glutamine in mitochondrial function and anabolic environment in cells: glutamine entering into cells is hydrolyzed to generate glutamate by glutaminase. Glutamine – leucine antiporter promotes the uptake of leucine, which activates mTORC1 and protein synthesis. Glutamate has multiple functions; uptake of cystine in exchange for glutamate promotes glutathione biosynthesis, which regulates ROS. ROS accumulation inhibits ATP production. Glutamate replenishes α-ketoglutarate (α-KG) to Krebs cycle and promotes ATP production and inhibits HIF1-α. ROS promotes non-enzymatic decarboxylation of α-KG to succinate, which is an activator of HIF1-α, glycolysis, and angiogenesis.

Activation of mTORC1 and creation of anabolic environment depend on ATP/ADP ratio (13). ATP production depends on glucose uptake. Studies on the relation between oxygen and glucose consumption (Crabtree effect) in the middle of twentieth Century [reviewed by Ibsen (23)] suggest that in response to glucose, cells consume initially high amounts of oxygen for about 20–120 s, which is followed by an inhibitory period but rises to stabilize around 30% of the original. Uncouplers of OXPHOS were shown to release the inhibitory effect on oxygen consumption, thus relating oxygen consumption and glucose uptake to ATP production. In terms of time frame, mitochondria devote only a short time (<2 min) for producing ATP in response to glucose availability and the buildup of ATP has regulatory effect on glucose uptake (24). ATP production, under OXPHOS, is 15-folds higher than in glycolysis and high ATP/ADP ratio is essential for functional stability of oncoprotein, Akt (25, 26) and to transform the intra cellular environment to ATP rich anabolic environment (Figure 1A).

## Glucose Uptake is Mediated by Akt S473 Which is Phosphorylated by mTORC2

Akt by phosphorylating its substrate AS160 was shown to mediate GLUT4 translocation to the membrane and promote glucose uptake. Interestingly, exercise/contractile activity also phosphorylates AS160 in muscle and AMP-activated protein kinase (AMPK) appears to have a role in this (27). Studies with rictor knock out cells had demonstrated that integrity of mTORC2 and phosphorylation of Akt S473 are essential for GLUT4 translocation and uptake of glucose (28, 29). Insulin resistance was shown to be the result of mTORC1 activation; one of its downstream targets, S6K, was shown to cause insulin resistance (30). Although phosphorylation of IRS1 by S6K was suggested to be the cause (31), recent studies demonstrated that S6k phosphorylation of rictor (32, 33) and mSin1 (34) results in mTORC2 inactivation. Further, inactivation of mTORC2 was shown to affect IIS mediated as well as PGDF and EGF mediated phosphorylation of Akt (34). This suggests that mTORC2 is upstream of Akt; it protects Akt during translation by phosphorylation of its turn motif site T450 and prevents its ubiquitination (22). Rapamycin inhibition of mTORC1 activates mTORC2 dependent AktS473. Rapamycin was also shown to up-regulate IGF-IR, Her2 expression, and reduced the phosphorylation of GSK-3β and NF-κB in an mTORC2 dependent way (35). These reports suggest that mTORC2 is up-regulated in metabolically starved or in mTORC1 inhibited cells and it is upstream of Akt activation.

## FoxO is the Regulator of Both mTOR and IIS

FoxO, as the transcription factor of rictor, plays a key role in the assembly of mTORC2; FoxO also inhibits mTORC1, but the inhibition depends on expression of Sestrin3, rictor, and activation of AMPK (36). FoxO3a is mainly activated by reactive oxygen species (ROS) and inhibits the mitochondrial gene expression (37). FoxO is the transcriptional regulator of insulin receptor (38), its substrate IRS-2 (39), and a reciprocal relation between FoxO and IRS was demonstrated in β-cells (40). In addition, it can inhibit Wnt pathway and proliferation of cells (41). FoxO also inhibits Myc and controls cell metabolism (42, 43).

## Aerobic Glycolysis, an Aberration on Mitochondrial Function

Warburg’s hypothesis of aerobic glycolysis centers on the respiratory damage (mitochondrial dysfunction) as the primary cause of malignancy. This, in our opinion, is an aberration of mitochondrial function. Traditionally mitochondria had been viewed as the ATP producing “power house” of the cell. It should be recognized that ATP production is only one of the short time functions of mitochondria. Substrate shuttles like, malate, aspartate, glutamate and citrate are critical for anaplerotic and cataplerotic reactions of Krebs cycle. They are the key sources of carbon and nitrogen requirements for protein and lipid biosynthesis of growing cells (5, 6). These reports suggest that mitochondria shift their function from ATP production to coordinate the biosynthetic function of proliferating cells and it has a time frame, which depends on ATP/ADP ratio and production of ROS in mitochondria. During active ATP production, ROS is kept under check and glutamine plays a critical role in redox homeostasis.

## Glutamine in Mitochondrial Metabolic Reprograming

Glutamine, avidly consumed by actively proliferating cancer cells (7, 8), has pleiotropic functions; chief amongst which are activation of mTORC1 signaling (44), regulation of glutamate levels, uptake of cysteine and leucine through the amino acids antiporters [reviewed in Ref. (8)]. Deamination of glutamine by two step reactions, glutaminase (GLS) and glutamate dehydrogenase, replenishes α-ketoglutarate (α-KG), which is critical for sensing oxygen, ATP production in transformed cells (Figure 1B). It is also a substrate for prolyl hydroxylases (PHDs), which inhibit HIF1α signaling (45, 46). ROS decarboxylate α-KG non-enzymatically to succinate (47) and inactivate PHDs and activate HIF1α signaling.

Glutaminase, which hydrolyzes glutamine to glutamate and ammonia, is up-regulated in several cancer cells (48, 49). Silencing of phosphate-activated mitochondrial GLS2 gene of HeLaR exposed to irradiation increased intracellular ROS and reduced the productions of antioxidants GSH, NADH, and NADPH (50). Glutamate exchange with cystine plays critical role in maintenance of the redox homeostasis mediated by cysteine thiol oxidation, thioredoxin system, and glutathione (GSH) peroxidases [reviewed in Ref. (51)].

Glutamine and glutamate are the substrates for leucine and cystine antiporters, while leucine is an activator of anabolism (52), cystine inside the cells is reduced to cysteine, which plays a key role in glutathione biosynthesis and redox homeostasis (53, 54). Interestingly, glutamine transport into cells is regulated by the inflammatory cytokine, the tumor necrosis factor α (TNF-α), which inhibits glutamine/Na^+^ co-transport (55) promotes the export of glutamate by activating cystine/glutamate transporters of microglia causes neurotoxicity in Japanese encephalitis (56). In contrast to cystine/glutamate transporter, activation of the leucine/glutamine antiporter SLC7A5/SLC3A2 and the amino acid sensor MAP4K3 improve leucine availability and were shown to activate mTORC1 and anabolic environment (57).

## The Lactic Acid Puzzle

Production of lactic acid in cancer cells is one of the strong arguments in favor of aerobic glycolysis (58). Lactate is produced from pyruvate in glycolysis; but in actively proliferating cells, oncogenes and ROS inhibit pyruvate kinase (PK) and block pyruvate production (59). Accumulated phosphoenolpyruvate (PEP) acts as a feedback regulator of glycolysis (60); altered glycolytic pathway supplies building blocks for biosynthesis of lipids and nucleic acids (61–63). It has been suggested in support of lactate theory, that cancer tissue is a mixture of catabolic and anabolic cells with variable access to oxygen and exhibits metabolic symbiosis; lactate produced in non-proliferating cells could be a source of fuel for proliferating cells (64, 65).

### Malate as a source of pyruvate

Mitochondrial substrate shuttles, like aspartate, glutamate, and succinate, which are key source of NADPH required for biosynthesis and the ATP, are also sources for malate [reviewed in Ref. (8, 45, 66)]. Malic enzyme (ME), which catalyzes the decarboxylation of malate to pyruvate exists in three isoforms, the cytosolic, mitochondrial NADP^+^ dependent forms, and the mitochondrial NAD^+^ dependent form (67, 68). ME plays a key role in lipogenesis and glutamine metabolism by generating the NADPH (69). Inhibition of MEs reciprocally activates p53, which regulates cell metabolism and proliferation (70). Anaplerotic flux of aspartate and glutamate in liver cells was shown to increase the lactate production (71); transamination of alanine to glutamate produces pyruvate, especially under glutamine deprived conditions.

In summary, we suggest that nitrogen source is critical to cell growth and oxygen’s role is to activate ATP production and limit glucose uptake. Nitrogen uptake and reduced dependency on glucose for growth in the presence of oxygen is the “Pasteur’s dictum.” Activation of mTORC1 by amino acids, independent of IIS, is the molecular recognition of “Pasteur’s dictum.” Activation of mTORC1 appears to precede mTORC2, which perhaps facilitates translation of Akt and its subsequent S473 phosphorylation and promotes glucose uptake and energy production as well as IIS activation. Stabilization of mTORC1 by IIS for anabolic activity of cells depends on ATP/ADP ratio in the absence of which, cells may recycle as stem cells. In anabolic environment, mitochondria reprogram their function for biosynthetic activity to replenish the carbon and amino acid resources for lipid and protein biosynthesis. Inhibition of mTORC1 in growing cells depends on ROS and AMPK. Higher levels of dietary amino acids result in longtime mTORC1 activation and inhibition of mTORC2 and insulin resistance, which appear to be the cause of metabolic pathologies and the secret of healthy life under dietary restriction (72).

## Abbreviations

Akt, protein kinase B (T308, S473 – phosphorylated sites threonine 308 and serine 473); AMPK, AMP-activated protein kinase; FoxO, fork head transcription factors of O group; GLUT, glucose transporter; GLS, Glutaminase; Her2, human epidermal growth factor receptor-2; IGF, insulin growth factor; IRS, insulin receptor substrate; IGF-IR, insulin-like growth factor-I receptor; mTORC1, 2, mechanistic target of rapamycin complex 1 and 2 (mTOR, formerly known as mammalian target of rapamycin); mLST8, MTOR associated protein, LST8 homolog (*S. cerevisiae*); mSIN1, mitogen-activated protein kinase associated protein 1; Myc, v-myc avian myelocytomatosis viral oncogene homolog (c-Myc); OXPHOS, oxidative phosphorylation; PEP, phosphoenolpyruvate; PA, phosphatidic acid; PDGF, platelet-derived growth factor; PI3FK, phosphatidylinositol 3-kinases; PIP2, phosphatidylinositol 4,5-bisphosphate; PIP3, phosphatidylinositol 3,4,5-trisphosphate; PLD1, phospholipase D1, phosphatidylcholine-specific ROS, reaction oxygen species; S6K, the p70 ribosomal, Sestrins, stress response proteins.
